# Aqueous solutions with information on solids: room-temperature phosphorescence of polysaccharide–benzophenone complexes†

**DOI:** 10.1039/d2ra08205e

**Published:** 2023-01-26

**Authors:** Masafumi Okuno, Keita Yamana, Riku Kawasaki, Yuto Konishi, Toshikazu Ono, Tsutomu Ishi-i, Atsushi Ikeda

**Affiliations:** a Applied Chemistry Program, Graduate School of Advanced Science and Engineering, Hiroshima University 1-4-1 Kagamiyama Higashi-Hiroshima 739-8527 Japan; b Department of Chemistry and Biochemistry, Graduate School of Engineering, Kyushu University 744 Motooka, Nishi-ku Fukuoka 819-0395 Japan; c Department of Biochemistry and Applied Chemistry, National Institute of Technology, Kurume College 1-1-1 Komorino Kurume 830-8555 Japan

## Abstract

Benzophenone and its derivatives emit crystallization-induced phosphorescence despite their simple structures. To easily modify their phosphorescence properties, we prepared phosphorescence-emitting aqueous solutions of polysaccharide–benzophenone and polysaccharide-4,4′-difluorobenzophenone complexes, which exhibit excellent biocompatibility and biodegradability.

## Abstract 2

Water-soluble λ-carrageenan–benzophenone complexes retained their properties of crystallization-induced phosphorescence of benzophenones at room temperature in aqueous solutions.

## Graphical abstract

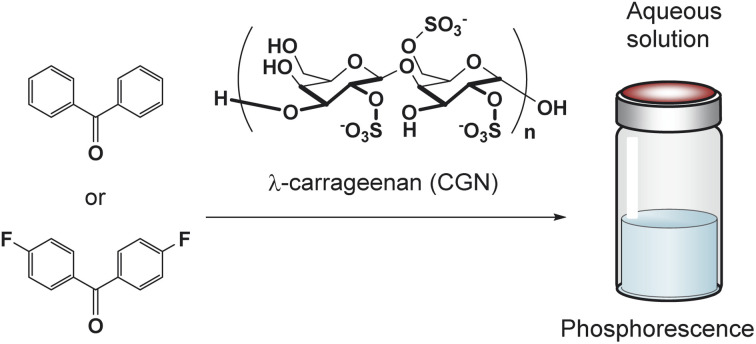

## Introduction

Since their discovery by Morantz *et al.*^1^ metal-free organic materials that emit room-temperature phosphorescence (RTP) have been receiving significant research interest.^2–7^ Most metal-free organic phosphorescent materials exist in the solid state at room temperature or in solution at a temperature below the melting point of the solvent.^1–7^ Recently, the formation of complexes with macrocyclic host molecules, such as cyclodextrins and cucurbiturils, or amphiphilic block copolymers has enabled the observation of organic phosphorescence in aqueous solutions at room temperature.^8–18^ These complexes in water are expected to be applied in bioimaging, exploiting their long emission lifetimes to avoid autofluorescence from endogenous fluorophores.^12,13^ Consequently, long-lived RTP materials improve signal-to-noise ratios because of the elimination of background interference by time-resolved imaging techniques.^19,20^ Previously, we succeeded in preparing aqueous solutions with information on solids (ASIS), such as aggregation-induced helical chirality in chiral crystals,^21^ crystal polymorphism,^22^ mechanochromic luminescence, and different fluorescence maxima depending on the crystal polymorphism^22^ or small aromatic guest molecules.^23^ In these studies, natural polysaccharides or polypeptides were used as the solubilizing agents because of their excellent biocompatibility and biodegradability.^24–26^ Therefore, RTP molecules in complexes with polysaccharides or polypeptides are expected to retain their phosphorescence properties in aqueous solutions.

**Fig. 1:**
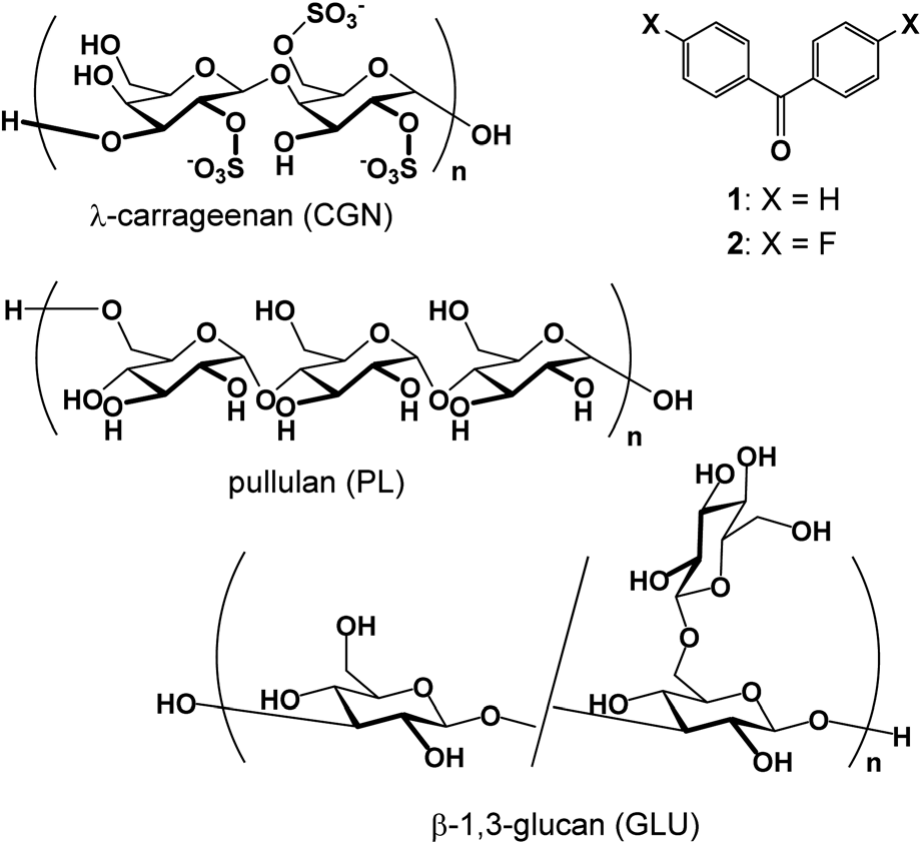
Structures of the compounds discussed in this study.

Benzophenone (1, Fig. 1) has been shown to emit phosphorescence at room temperature in the solid state.^27,28^ The phosphorescence in the crystal form is centered at 449 nm, with an average lifetime of 317 μs.^27^ Conversely, 4,4′-difluorobenzophenone (2, Fig. 1) has a longer phosphorescence lifetime (〈*τ*〉) and higher quantum yield (*Φ*) than 1 (2: 〈*τ*〉 = 1297 μs, *Φ* = 40%; 1: *Φ* = 16%).^27^ In this study, these benzophenones were solubilized in water using polysaccharides, such as pullulan (PL), λ-carrageenan (CGN), and β-(1,3-1,5)-d-glucan (GLU) as the solubilizing molecules, and they retained their phosphorescence properties (Fig. 1). Using different solubilizing molecules and methods, we investigated the solubilizing ability, phosphorescence properties, and stability of the aqueous solutions.

## Results and discussion

After the crystallization of 1 and 2 from toluene, each crystal (5.0 μmol) was mixed with CGN (10 mg) using a mechanochemical high-speed vibration milling (HSVM) technique.^21,29,30^ Similarly, the crystals of 1 or 2 were mixed with CGN, PL, or GLU (10 mg) by grinding using a porcelain mortar and pestle.^22^ Thereafter, these solid mixtures were extracted using ultrapure water, and the resulting resin was separated from the aqueous solution by centrifugation for 20 min at 4500 rpm.

**Fig. 2:**
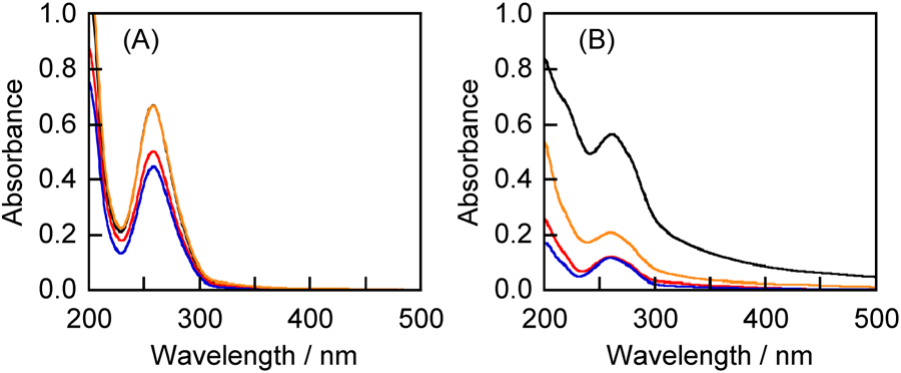
UV-vis absorption spectra of the aqueous solutions of (A) the CGN–1 complex (black) prepared by the HSVM method and the CGN–1 (red), PL–1 (blue), and GLU–1 (orange) complexes prepared by the grinding method; (B) the CGN–2 complex (black) prepared by the HSVM method and the CGN–2 (red), PL–2 (blue), and GLU–2 (orange) complexes prepared by the grinding method (1 mm cell, 20 °C).

All the aqueous solutions of 1 complexed with polysaccharides exhibited the absorption maximum at 257 nm, indicative of the absorption of 1 (Fig. 2A). Consequently, 1 could be solubilized by the three polysaccharides in both the HSVM and grinding methods. The concentrations of 1 in the aqueous solutions were determined based on the molar extinction coefficient after adding methanol (water : methanol = 1 : 9 (v/v)) to disrupt the complexes. The calculated concentrations of 1 in the CGN–1 complexes prepared by the HSVM and the grinding methods are 0.93 and 0.70 mmol L^−1^, respectively. The result indicates that the solubility of 1 in the grinding method was comparable to that in the HSVM method. In contrast, the concentration of 1 in the GLU–1 complex (0.93 mmol L^−1^) was higher than those in the CGN–1 and PL–1 complexes prepared by the grinding method (0.70 and 0.62 mmol L^−1^, respectively).

The appearance of the absorption maximum at 261 nm for all the aqueous solutions of the CGN–2, PL–2, and GLU–2 complexes indicates that the fluorinated benzophenone (2) in all the complexes was dissolved in water (Fig. 2B). The concentrations of 2 in the CGN–2 complexes prepared by the HSVM and the grinding methods are 2.52 and 0.54 mmol L^−1^, respectively. The solubilities of the polysaccharide–2 complexes prepared by the HSVM method were considerably higher than those of the complexes prepared by the grinding method. The concentration of 2 in the GLU–2 complex (0.93 mmol L^−1^) was higher than those in the CGN–2 and PL–2 complexes prepared by the grinding methods (0.54 and 0.52 mmol L^−1^, respectively).

In summary, the solubilities of the polysaccharide–1 and –2 complexes prepared by the grinding method are in the order of GLU– > CGN– ≈ PL–(GLU–1: 0.93, CGN–1: 0.70, and PL–1: 0.62 mmol L^−1^, GLU–2: 0.93, CGN–2: 0.54, and PL–2: 0.52 mmol L^−1^). The polysaccharide–1 complexes exhibited higher solubilities than the polysaccharide–2 complexes.

**Fig. 3:**
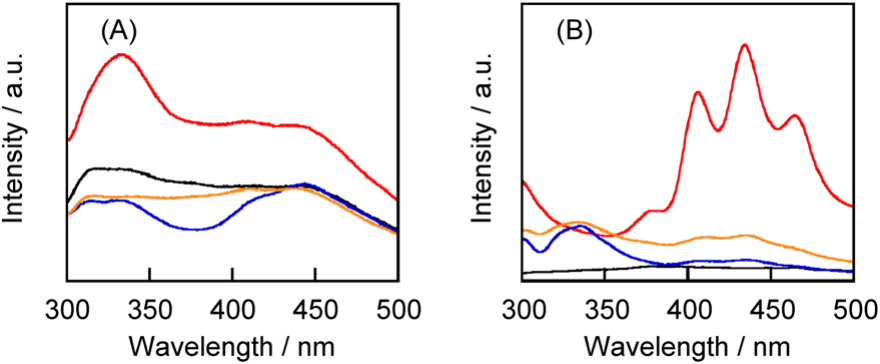
Fluorescence spectra of the aqueous solutions of (A) the CGN–1 complex (black) prepared by the HSVM method and the CGN–1 (red), PL–1 (blue), and GLU–1 (orange) complexes prepared by the grinding method; (B) the CGN–2 complex (black) prepared by the HSVM method and the CGN–2 (red), PL–2 (blue), and GLU–2 (orange) complexes prepared by the grinding method (1 cm cell, 20 °C). (A) *λ*_ex_ = 270 nm, ex/em slits = 20/20 nm; (B) *λ*_ex_ = 270 nm, ex/em slits = 10/10 nm.

Although the solubility of 1 in the polysaccharide–1 complexes was not significantly influenced by the mixing method and type of polysaccharide, the aqueous solutions exhibited considerably different fluorescence and phosphorescence spectra (Fig. 3A). The aqueous solution of the CGN–1 complex prepared by the grinding method exhibited higher-intensity fluorescence and phosphorescence peaks in the 300–375 and 400–500 nm regions, respectively,^27^ compared with those for the other solutions (Fig. 3A). In particular, the GLU–1 complex exhibited weak emission despite its high solubility. This result suggests that the CGN–1 complex prepared by the grinding method contains the highest proportion of 1 in the solid state. However, three barely visible peaks assignable to the phosphorescence of 1 in the crystalline state at room temperature were observed at 420, 449, and 483 nm.^24^ In contrast, the aqueous solution of the CGN–2 complex exhibited higher-intensity fluorescence and phosphorescence peaks compared with those for the other solutions of 2 (Fig. 3B). Furthermore, the fluorescence and phosphorescence peak intensities for the aqueous solution of the CGN–2 complex were considerably higher than those for the CGN–1 complex under the same conditions ([1] = [2] = 0.50 mM) (Fig. S1†). The peaks at 406, 435, and 465 nm for the CGN–2 complex are similar to those at 409, 436, and 467 nm of 2 observed in the crystalline state at room temperature.^27^ Therefore, we used the CGN–2 and CGN–1 complexes as the reference for all subsequent experiments.

**Fig. 4:**
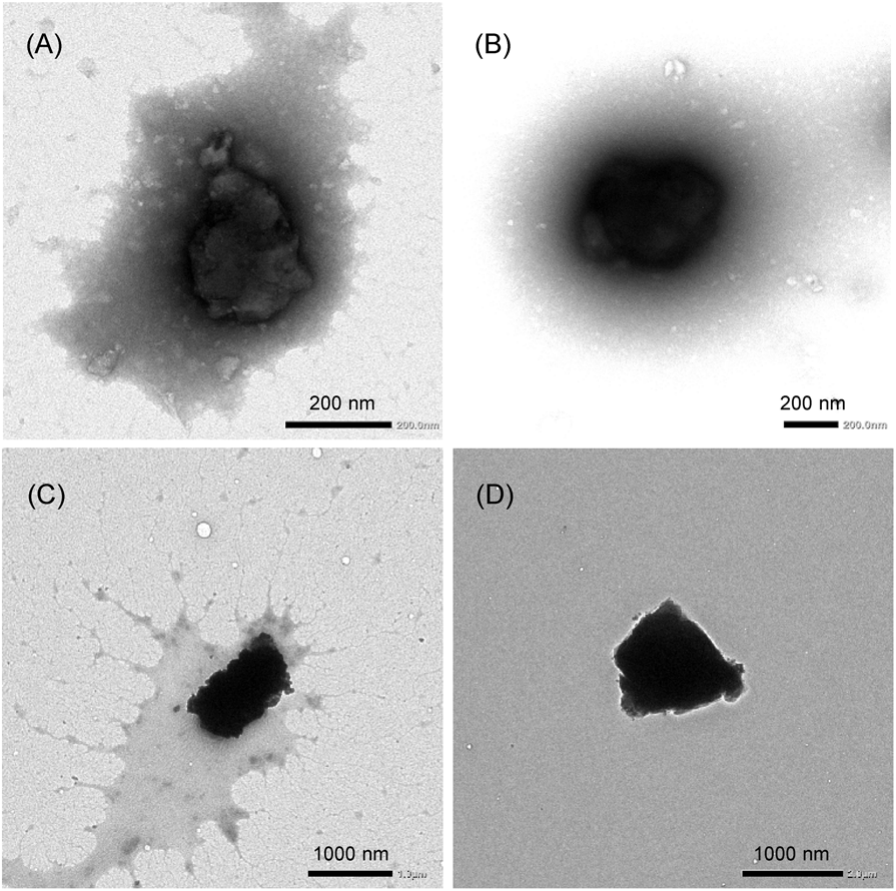
TEM images of the (A) CGN–1 and (B) CGN–2 complexes prepared by the HSVM method and the (C) CGN–1 and (D) CGN–2 complexes prepared by the grinding method (negative staining, CGN–1: 2.0 wt% phosphotungstic acid, CGN–2: 3.0 wt% ammonium molybdate).

The hydrodynamic diameters (*D*_hy_) of the CGN–1 and CGN–2 complexes prepared by the HSVM method were determined to be 299 and 352 nm, with polydispersity indexes of 0.17 and 0.22, respectively, by dynamic light scattering (DLS) measurements (Table 1 and Fig. S2†). In contrast, the *D*_hy_ values of the CGN–1 and CGN–2 complexes prepared by the grinding method (1058 and 1519 nm, respectively) were considerably larger than those prepared by the HSVM method. Furthermore, the morphologies of the CGN–1 and CGN–2 complexes were observed by transmission electron microscopy (TEM). As shown in Fig. 4A and B, globular structures with diameters of approximately 300 nm were observed in both the CGN–1 and CGN–2 complexes prepared by the HSVM method. Furthermore, globular structures with diameters of approximately 1000 nm were observed in both the CGN–1 and CGN–2 complexes prepared by the grinding method (Fig. 4C and D). These diameters were consistent with the *D*_hy_ values determined by DLS measurements (Table 1).

**Table 1:** Average hydrodynamic diameters (*D*_hy_/nm) of the CGN–1 and CGN–2 complexes prepared by the HSVM and the grinding methods in water

Complex	Preparation method	*D*_hy_/nm^a^	PDI^a^^,^^b^
CGN–1	HSVM	299 ± 10	0.17
CGN–2	HSVM	352 ± 10	0.22
CGN–1	Grinding	1058 ± 44	0.20
CGN–2	Grinding	1518 ± 33	0.46

a The *D*_hy_ values were determined by DLS measurements in Milli-Q water (25 °C).

b The PDI was calculated using the cumulant method.

**Fig. 5:**
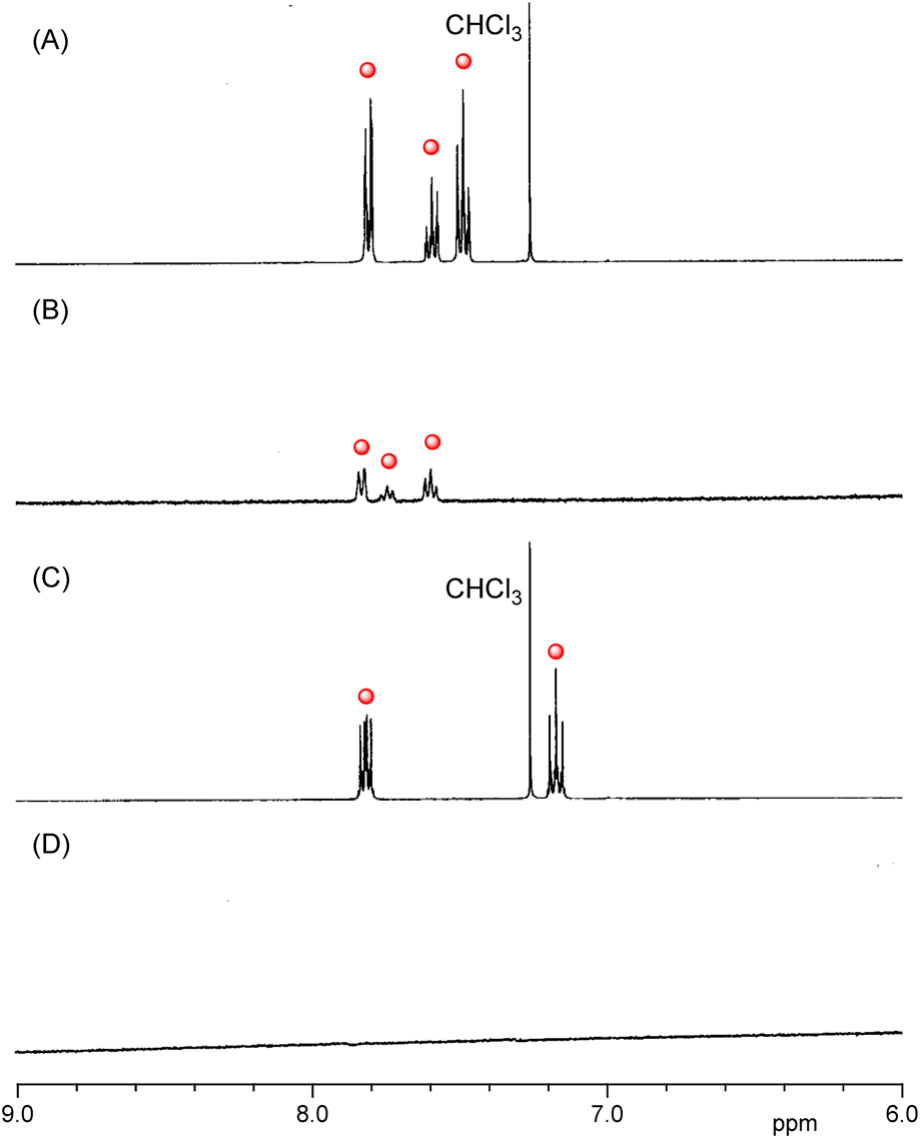
Partial ^1^H NMR spectra of (A) 1 in CDCl_3_, (B) the CGN–1 complex in D_2_O, (C) 2 in CDCl_3_, and (D) the CGN–2 complex in D_2_O (400 MHz, 20 °C). Red circles show the peaks assignable to 1 or 2. These complexes were prepared by the grinding method.

To ascertain whether 1 or 2 leaked from their respective CGN complexes, proton nuclear magnetic resonance (^1^H NMR) spectra were measured (Fig. 5 and S3†). All the peaks of the guest molecules in the large self-aggregate disappeared because of peak broadening. However, the peaks of the free guest molecules leaked from the complexes appeared in the ^1^H NMR spectra.^29–33^ In the D_2_O solution of the CGN–1 complex, peaks assignable to 1 appeared at the 7.5–8.0 ppm region (Fig. 5B), attributed to the slight water solubility of 1 (0.14 g L^−1^). The result indicates that several portions of 1 leaked from the CGN–1 complex. The leakage percentage of 1 from the CGN–1 complex was estimated to be 4%, based on the peak intensity of 1 relative to the peak intensity of DMSO added as the internal reference. In contrast, no peaks of 2 in the CGN–2 complex appeared at the 7.0–8.0 ppm region, indicating that most of 2 were incorporated in the complex (Fig. 5D).

**Fig. 6:**
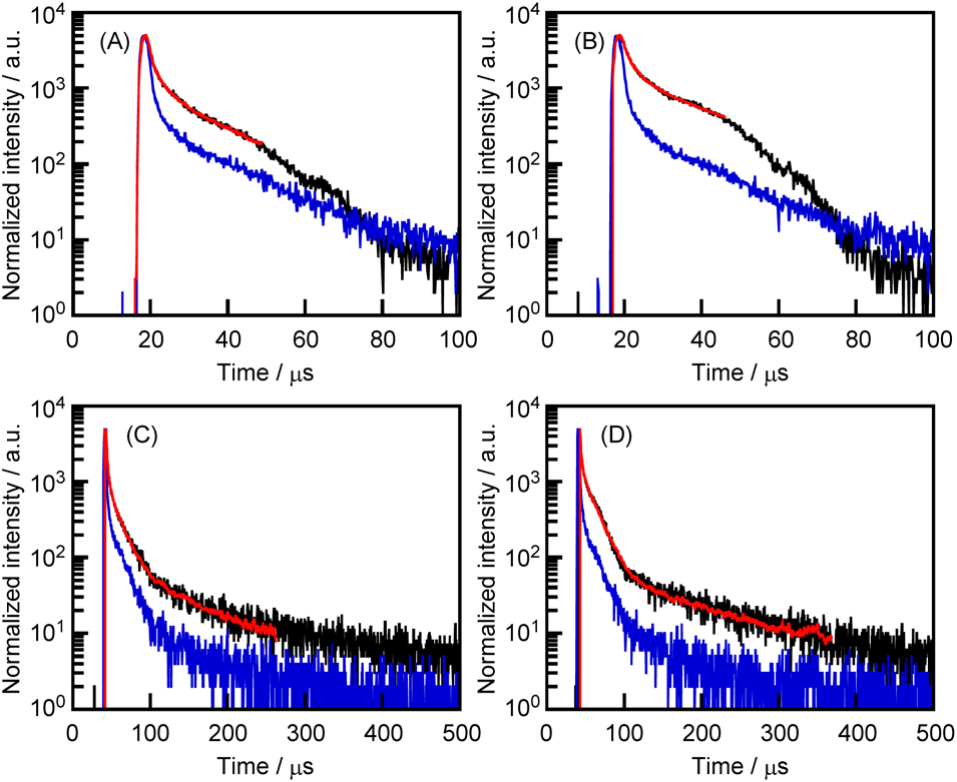
Time-resolved photoluminescence decay curves (black), fits (red), and instrument response function (blue line) for the phosphorescence at 400 nm of (A) the CGN–1 and (B) the CGN–2 complexes prepared by the HSVM method and at 440 nm of (C) the CGN–1 and (D) the CGN–2 complexes prepared by the grinding method in H_2_O at room temperature.

In Fig. 3, the peaks at the 400–500 nm region are predicted to be indicative of phosphorescence. Therefore, the photoluminescence spectral decay behavior of the CGN–1 and CGN–2 complexes was investigated by a time-resolved technique (Fig. 6). To fit the fluorescence decays (red lines), we measured the system's temporal instrument response function (blue lines). The measurement data were subtracted from the time-resolved photoluminescence decay curve (black lines) data to compensate for the distortions in the data caused by the instrument response function (Fig. 6). The emissions of the CGN–1 and CGN–2 complexes decay through three relaxation pathways; the same applies to the crystallization-induced phosphorescence of 1 and 2. The mean lifetimes of the CGN–1 and CGN–2 complexes prepared by the grinding method (〈*τ*〉 = 10.6 and 29.7 μs, respectively) were longer than those of the complexes prepared by the HSVM method (〈*τ*〉 = 4.7 and 8.3 μs, respectively) (Fig. 6 and Table 2). In contrast, the mean lifetime of the CGN–2 complex prepared by the grinding method (〈*τ*〉 = 29.7 μs) was longer than that of the CGN–1 complex prepared by the grinding method (〈*τ*〉 = 10.6 μs) (Fig. 6 and Table 2). These results clearly indicate that the CGN–1 and CGN–2 complexes retained the property of crystallization-induced phosphorescence of 1 and 2 in the aqueous solutions. However, the 〈*τ*〉 values of 1 and 2 in the complex state were considerably lower than those in the solid state (317 and 1297 μs, respectively) (Fig. 6).^27^

**Table 2:** Phosphorescence properties of the aqueous solutions of the CGN–1 and CGN–2 complexes prepared by the HSVM and grinding methods (*λ*_ex_ = 280 nm)

Preparation method		*λ*_RTP_ (nm)	Phosphorescence decay			
			*τ*_1_/μs (*A*_1_/%)	*τ*_2_/μs (*A*_2_/%)	*τ*_3_/μs (*A*_3_/%)	〈*τ*〉/μs
CGN–1	HSVM	400	0.1 (97.3)	2.3 (2.0)	11.1 (0.6)	4.7
CGN–2	HSVM	400	0.1 (90.6)	2.0 (7.4)	15.1 (1.9)	8.3
CGN–1	Grinding	440	0.7 (94.8)	6.9 (5.0)	63.5 (0.2)	10.6
CGN–2	Grinding	440	1.0 (93.2)	11.0 (6.7)	188.8 (0.1)	29.7

## Experimental

### Materials

Benzophenone (1) and 4,4′-difluorobenzophenone (2) were purchased from Kanto Chemical Co., Ltd. (Tokyo, Japan). Pullulan (PL) and λ-carrageenan (CGN) were procured from Tokyo Chemical Industry Co., Ltd. (Tokyo, Japan) and FUJIFILM Wako Pure Chemical Corporation (Osaka, Japan), respectively. β-(1,3-1,5)-d-Glucan (GLU) was used after purification using Aureobasidium pullulans black yeast.

### Preparation of the CGN–1 and CGN–2 complexes by the HSVM method

Compound 1 or 2 (1.0 mg, 5.0 and 4.6 μmol, respectively) and CGN (10.0 mg) were placed in an agate capsule with two agate-mixing balls and mixed vigorously at 30 Hz for 20 min using a high-speed vibration mill (MM 200; Retsch Co., Ltd., Haan, Germany). The solid mixture was suspended in water (2.0 mL) to produce a white emulsion. After centrifugation (4500×*g*, 25 °C, 20 min), the undispersed 1 or 2 materials were removed from the solution. The concentrations of 1 and 2 in the complexes with CGN were determined by the absorbances at 254 and 256 nm, respectively, of the aqueous solutions (0.1 mL) of the CGN–1 and CGN–2 complexes diluted by methanol (0.9 mL). The Abs_254_ and Abs_256_ values in water : methanol = 1 : 9 (v/v) solutions for the CGN–1 and CGN–2 complexes were 0.6837 and 0.2606 (1 mm cell). As the molar absorption coefficients of 1 and 2 in water : methanol = 1 : 9 (v/v) were *ε*_254_ = 6.83 × 10^4^ dm^3^ mol^−1^ and *ε*_256_ = 1.53 × 10^4^ dm^3^ mol^−1^, respectively, the concentrations of 1 and 2 in water : methanol = 1 : 9 (v/v) were determined to be 9.81 × 10^−5^ and 1.18 × 10^−4^ M, respectively, by the Beer–Lambert law. Therefore, the concentrations and molar absorption coefficients of 1 and 2 in the aqueous solutions of the CGN–1 and CGN–2 complexes were also 9.81 × 10^−4^ M and 1.18 × 10^−3^ M and *ε*_254_ = 7.00 × 10^3^ dm^3^ mol^−1^ and *ε*_256_ = 2.21 × 10^3^ dm^3^ mol^−1^, respectively, by the Beer–Lambert law.

### Preparation of the polysaccharide–1 and –2 complexes by the grinding method

Compound 1 or 2 (1.0 mg; 5.0 and 4.6 μmol, respectively) and CGN, PL, or GLU (10.0 mg) were placed in a ceramic mortar. The mixture was prepared by mild grinding for 30 min. The solid mixture was suspended in water (2.0 mL) to produce a white emulsion. After centrifugation (4500×*g*, 25 °C, 20 min), undispersed 1 or 2 was removed from the solution. The concentrations of 1 and 2 in the complexes with CGN, PL, or GLU were determined by using the same *ε*_254_ and *ε*_256_ of the aqueous solutions of the CGN–1 and CGN–2 complexes prepared by the HSVM method. The concentrations of 1 or 2 in the aqueous solution of the CGN–1, PL–1, GLU–1, CGN–2, PL–2, and GLU–2 complexes were determined to be 0.93, 0.70, 0.62, 0.93, 0.54, and 0.52 mmol L^−1^, respectively.

### Spectrophotometric assay

The absorbance spectra were recorded using a UV-3600 spectrophotometer (Shimadzu Corporation, Kyoto, Japan). The fluorescence spectra were obtained using an F-4500 fluorescence spectrophotometer (Hitachi Ltd, Tokyo, Japan). The excitation and emission wavelengths were set to 270 and 300–500 nm, respectively. The time-resolved photoluminescence lifetimes were determined using a time-correlated single photon counting lifetime spectroscopy system, Quantaurus-Tau C11367-02 (Hamamatsu photonics K. K., Shizuoka, Japan).

### Dynamic light scattering analysis

The hydrodynamic diameters and zeta potentials of the liposomes were measured using an electrophoretic light-scattering instrument, equipped with a laser Doppler system (Zetasizer Nano ZS, Malvern Instruments Ltd., Malvern, UK).

### Transmission electron microscopy

The 1−PLL complex was assessed by TEM using the negative staining method with ammonium molybdate (3.0 wt%). A solution of the complex was cast on a Cu100P grid coated with collodion films (thickness: 30–40 nm) supported by a carbon layer (thickness: 10–15 nm). The grid was pre-treated with low-energy plasma (DII-29020HD, JEOL Ltd., Tokyo, Japan) to hydrophilize the surface. The extra solution was removed using filter paper and subsequently air-dried. TEM observations were performed using the JEM-1400 system (JEOL Ltd., Tokyo, Japan) with an accelerating voltage of 80 kV.

### Lifetime measurements

The following two exponential emission decay models were applied to fit the whole image:
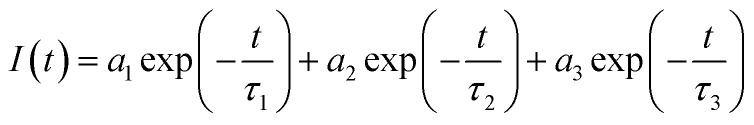
where *I*(*t*) is the emission counts that were collected at time *t* after excitation; *a*_1_, *a*_2_, and *a*_3_ represent the corresponding amplitudes; and *τ*_1_, *τ*_2_, and *τ*_3_ are the lifetimes for the short and long components. An average emission lifetime, 〈*τ*〉, defined by the following equation was calculated:
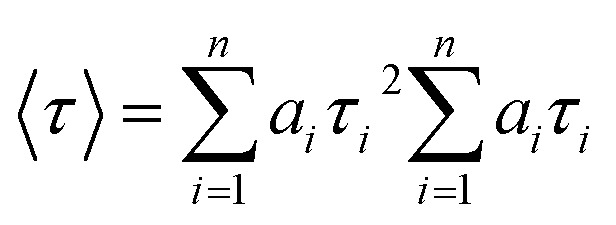


The quality of the nonlinear curve fit was determined and statistically measured using the minimum reduced chi-squared (*χ*^2^). A value of 1.0 corresponds to a perfect fit.

## Conclusions

We succeeded in preparing ASIS with crystallization-induced RTP using both the CGN–1 and CGN–2 complexes. The mean lifetime 〈*τ*〉 of the CGN–2 complex was longer than that of the CGN–1 complex. This was due to the solid state and the long-life of the triplet state, attributed to the inhibition of intramolecular motions (*e.g.*, rotational and vibrational energy relaxation) by the intramolecular interactions of C–H⋯F.^27^ However, the 〈*τ*〉 values of the complexes were considerably shorter than those of 1 and 2 in the solid state. Therefore, compounds with longer RTP lifetimes should be employed to achieve extended emissions in the future.

## Conflicts of interest

There are no conflicts to declare.

## Supplementary Material

RA-013-D2RA08205E-s001
