# Supplementary material for: Aqueous solutions with information on solids: room-temperature phosphorescence of polysaccharide–benzophenone complexes
Source: RSC Adv. 2023 Jan 26;13(6):3528–33. doi: 10.1039/d2ra08205e (PMC9891091; doi:10.1039/d2ra08205e)
Supplement: RA-013-D2RA08205E-s001 [file RA-013-D2RA08205E-s001.pdf]

Electronic Supplementary Material (ESI) for RSC Advances.
This journal is © The Royal Society of Chemistry 2023

Electronic Supplementary Information

Aqueous solutions with information on solids: room-temperature phosphorescence of polysaccharide–benzophenone complexes

Masafumi Okuno,[a] Keita Yamana,[a] Riku Kawasaki,[a] Yuto Konishi,[b] Toshikazu Ono,[b] Tsutomu Ishi-i[c] and Atsushi Ikeda[a,*]

[a]Applied Chemistry Program, Graduate School of Advanced Science and Engineering, Hiroshima University, 1-4-1 Kagamiyama, Higashi-Hiroshima 739-8527, Japan.

[b]Department of Chemistry and Biochemistry, Graduate School of Engineering, Kyushu University, 744 Motooka, Nishi-ku, Fukuoka 819-0395, Japan.

[c]Department of Biochemistry and Applied Chemistry, National Institute of Technology, Kurume College, 1-1-1 Komorino, Kurume 830-8555, Japan.

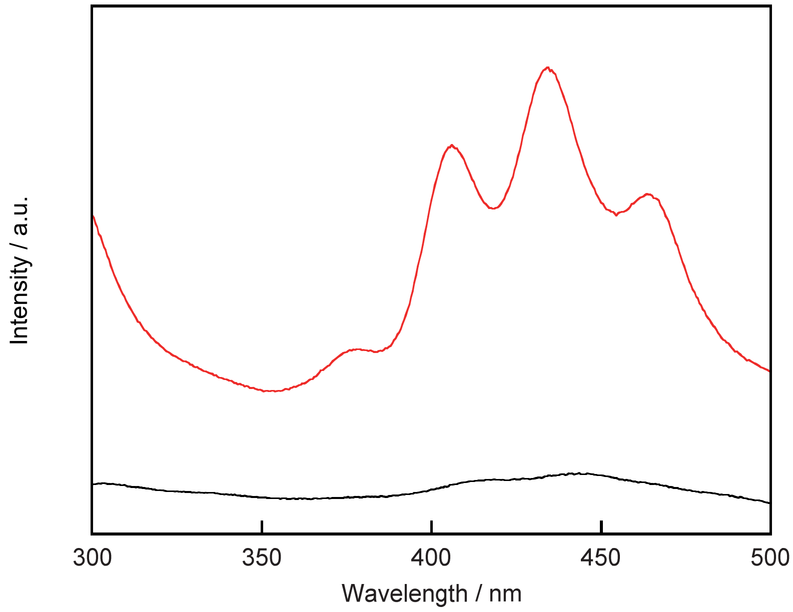

Fig. S1 Fluorescence spectra of the aqueous solutions of the CGN–**1** (black) and CGN–**2** (red) complexes prepared by the grinding method (1 cm cell, 20 °C). λ_{ex} = 270 nm, ex/em slits = 10/10 nm, [**1**] = [**2**] = 0.50 mM.

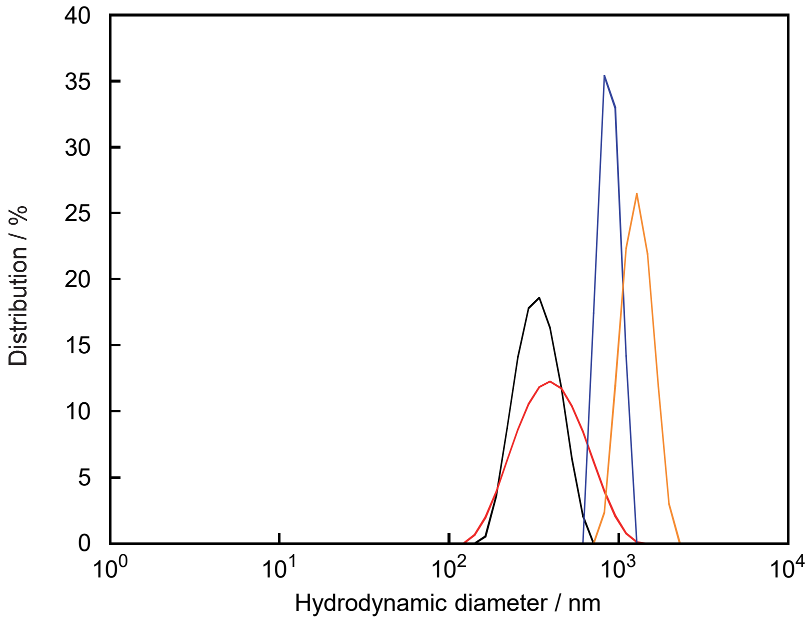

Fig. S2 DLS size-distribution profile for the aqueous solutions of the CGN–**1** (black) and CGN–**2** (red) complexes prepared by the HSVM method and the CGN–**1** (blue) and CGN–**2** (orange) complexes prepared by the grinding method.

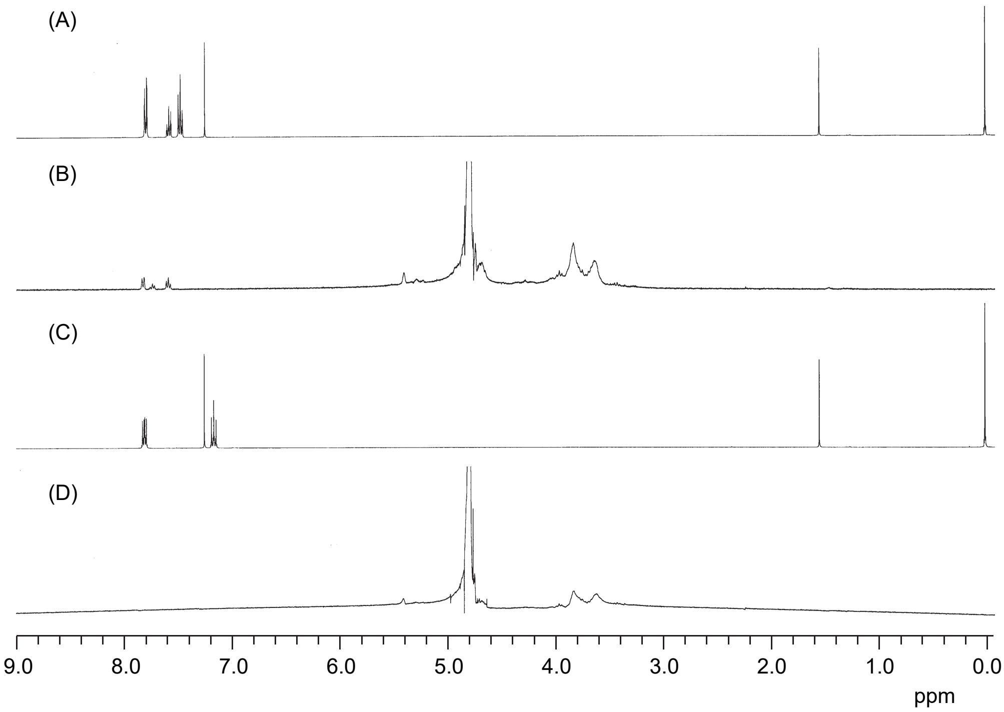

Fig. S3 ^{1}H NMR spectra of (A) **1** in $CDCl_3$, (B) the CGN–**1** complex in D_2O, (C) **2** in $CDCl_3$, and (D) the CGN–**2** complex in D_2O (400 MHz, 20 °C). These complexes were prepared by the grinding method.

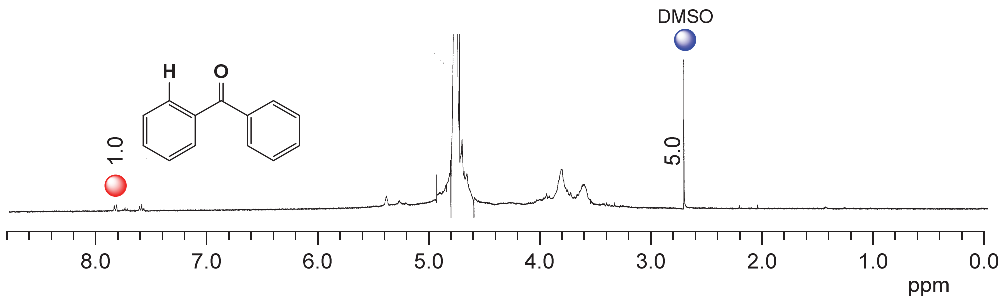

Fig. S4 ^{1}H NMR spectra of the CGN–**1** complex in D_2O with DMSO as the internal standard (400 MHz, 20 °C, [DMSO] = 0.145 mM). The complex was prepared by the grinding method.
